# Who is dying from COVID-19 in the United Kingdom? A review of cremation authorisations from a single South Wales' crematorium

**DOI:** 10.1017/S0950268821000054

**Published:** 2021-01-08

**Authors:** R. L. Salmon, S. P. Monaghan

**Affiliations:** Cardiff Thornhill Crematorium, Thornhill, Cardiff CF14 9UA, UK

**Keywords:** Coronavirus, COVID-19, deaths (hospital and community), hospital-acquired (nosocomial) infections, public health

## Abstract

Only studies in the UK on individuals dying from coronavirus disease 2019 (COVID-19) in hospital have been published, to date. Cremation law requires collection of clinical information that can improve understanding of deaths in both hospital and community settings. Age, sex, date and place of death, occupation, comorbidities and where infection acquired was recorded for all deaths from COVID-19, between 6 April and 30 May, for whom an application was made for cremation at a South Wales' crematorium. Of 752 cremations, 215 (28.6%) were COVID-19 (115 (53.5%) male and 100 (46.5%) female). Median age was 82 years (youngest patient 47 and the oldest 103 years). Over half the deaths (121/215: 56.3%) were over 80 years. Males' odds of dying in hospital, rather than the community were 1.96 times that of females (95% confidence intervals (CI) 1.03–3.74, *P* = 0.054) despite being of similar age and having a similar number of comorbidities. Only 21 (9.8%) of 215 patients had no comorbidities recorded. Patients dying in care homes were significantly older than those dying in hospital (median 88 years (interquartile (IQ) range 82–93 years) *vs.* 80 years (IQ range 71–87 years): *P* < 0.0001). Patients dying in hospital had significantly more comorbidities than those dying in care homes (median 2: IQ range 1–3 *vs.* 1: IQ range 1–2: *P* < 0.001). Sixty three (29.3%) of infections were hospital acquired and a further 55 (25.6%) acquired in care homes. In a series, of hospital and community deaths, persons over 80 with an average two comorbidities predominated. Men were more likely to die in hospital. Half the infections were acquired in hospitals or care homes with implications for management of the pandemic.

## Introduction

Coronavirus disease 2019 (COVID-19), a viral infectious disease caused by the severe acute respiratory syndrome-coronavirus-2 (SARS-CoV-2), emerged in China by the end of 2019 and was declared pandemic by the World Health Organisation on 11 March 2020 [1]. The first case occurred in the UK on 31st January 2020 and in Wales, where 1441 deaths have occurred as of 12 June 2020 [2], on 28th February 2020 [3]. The disease has given rise to a number of far-reaching public health measures. So far, in the UK, there have been three large community studies, of which in two, the main outcome is deaths in hospital [4, 5] and in the third positive SARS-CoV-2 tests [6]. There has also been a small study of risk factors for intensive care unit admissions in South Wales [7]. All identify similar risks for mortality and morbidity; increasing age, male gender, diabetes, chronic heart, lung, kidney or neurological disease, malignancy and dementia. Importantly, although there has been an ecological study comparing death rates, including out-of-hospital deaths, by local authority area, in Great Britain [8], no UK studies, to date, have reported the characteristics and risks of death in individuals dying from COVID-19 that includes those patients dying out-of-hospital, in care homes or at home, as well as those dying in hospital. This is significant because little is known about the personal or clinical characteristics of cases that may make them more likely to be admitted to hospital and therefore to what extent in-hospital deaths reflect deaths from COVID-19 as a whole.

In 2012, as a result of The Cremation (England and Wales) Regulations 2008 [9], themselves arising out of the Inquiry into Harold Shipman, a doctor and serial murderer [10], a new suite of forms for the authorisation of cremation came into use. Although procedures have been temporarily simplified to assist the management of the Covid-19 pandemic [11], the key information requirements are unchanged. The cause of death is recorded by a doctor who has attended the patient (criteria for attendance are defined in the legislation [11]) and is identically worded to the Medical Certificate of the Cause of Death (MCCD). This wording is largely at the clinical discretion of the doctor in attendance and need not use codes from the International Classification of Diseases (ICD), although national guidance for certifying doctors exists [12]. Cremation authorisation rules emphasise that the fact and cause of death has been definitely ascertained and require a brief text account (question 9, form 4) of the ‘symptoms and other conditions’ that led to the conclusions about the cause of death, as recorded on the form. This account, although unstructured, permits a view of the course of the final illness which can be used better to understand deaths from COVID-19.

Cardiff Thornhill Crematorium is a Local Authority run crematorium (run by Cardiff Council, one of 22 unitary local authorities in Wales) that performed 2850 cremations in 2017, a typical year in terms of activity.

In order better to inform public health policy, those individuals dying from COVID-19, that were authorised for cremation, were characterised in terms of age, sex, occupation, comorbidities and where the infection had been acquired.

## Methods

Information was taken from: Cremation Form 4 in which a certifying clinician is required both to record the cause of death (question 11) in a format that reflects the separate MCCD and to give a brief account (question 9) of the ‘symptoms and other conditions’ that led them to that conclusion: Cremation Form 1 in which the applicant for cremation (usually the next of kin) gives the age and occupation of the deceased. Both forms record the home address, date and time of death. Ethnicity, which is a well-known risk factor for death from COVID-19, however, is not recorded.

Deaths were defined as COVID-19 if specifically mentioned in response to q.11 or if a SARS-CoV-2 positive test was documented in response to q.9. Comorbidities were any relevant conditions mentioned in answer to either of questions 9 or 11.

The authors authorised the cremations of all patients who were cremated at Cardiff Thornhill crematorium over the study period. Data were entered onto a structured *pro forma;* sex, age, occupation, date of death, location of death (hospital, care home, own home), comorbidities and whether infection was acquired in hospital (defined as occurring 6 days after admission OR following admission for an unrelated condition). Care home residents were assumed to have acquired their infection in their care home unless they fulfilled the criteria for having acquired the infection in hospital.

Analysis was performed in EpiInfo version 7(US Centers for Disease Control and Prevention (CDC)) [13]. Median and interquartile (IQ) ranges were calculated for age and the number of comorbidities; simple frequencies for other variables. Differences in patient characteristics and comorbidities (i) between men and women and (ii) by place of death were examined using the Mann−Whitney *U* test for continuous variables and the chi-squared test (Yates corrected) for categorical variables.

## Results

**Fig. 1.**
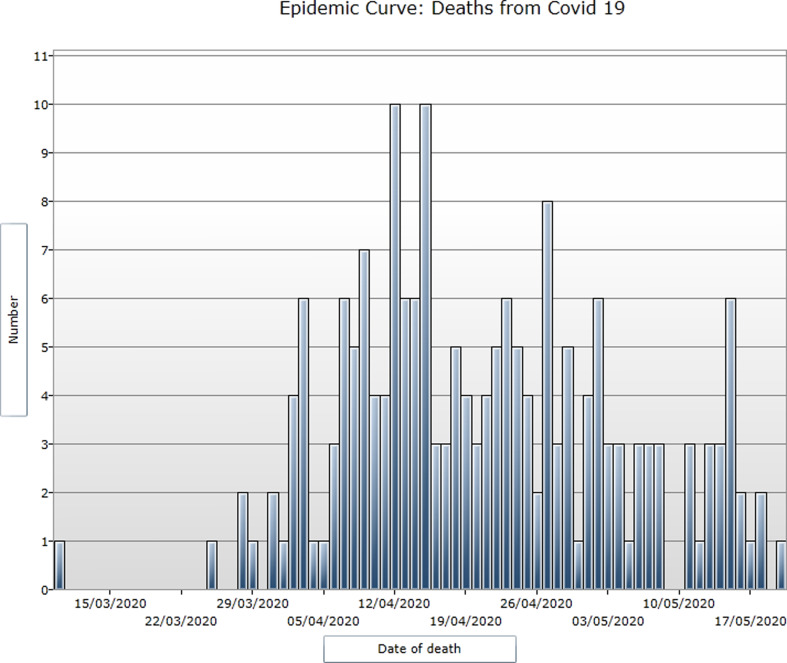
Epidemic curve: dates of death of individuals with COVID-19.

Of the total of 752 cremations authorised over the period of 6th April until 29th May, 215 (28.6%) were COVID-19 of which 115 (53.5%) were male and 100 (46.5%) female (*P* = NS). Dates of death are shown in Figure 1. The median age was 82 years, with the youngest patient being 47 years and the oldest 103 years. Over half the deaths (121/215: 56.3%) were over 80 years. Among 146 patients who died in hospital, 85 (58.2%) were male and 61 (41.8%) were female (*P* NS). Males' odds of dying in hospital, rather than the community were 1.96 times that of females (95% confidence intervals (CI) 1.03–3.74, *P* = 0.054) despite being of similar age (males: median age 81 years: IQ range 72−87 years *vs.* females: 84 years: IQ range 72–90.5 years) and having a similar number of comorbidities (both sexes: median 2, range 0–7). Only 21 (9.8%) of 215 patients had no comorbidities recorded.

Patients' comorbidities, by place of death, are shown in Table 1. Patients dying in care homes were significantly older than those dying in hospital (median 88 years (IQ range 82–93 years) *vs.* 80 years (IQ range 71–87 years): *P* < 0.0001). Patients dying in hospital had significantly more comorbidities than those dying in care homes (median 2: IQ range 1–3 *vs.* 1: IQ range 1–2: *P* < 0.001). Patients dying in hospital were significantly more likely than those dying in care homes, to have diagnoses of chronic heart disease (*P* < 0.05) or chronic pulmonary disease, excluding asthma (*P* < 0.05) but significantly less likely of dementia (*P* < 0.000005). No patient dying in their own home had a diagnosis of dementia (*P* < 0.001).

**Table 1.** Characteristics and comorbidities in patients dying of Covid-19, by place of death

Characteristics	All	Hospital	Care home	Own home	*P*
Study subjects	215	146	43	10	**–**
Age (years, median, IQ range)	82 (72**–**89)	80 (71**–**87)	88 (82**–**93)	75 (68**–**91)	<0.0001^a^
40**–**49 years	2 (0.9%)	1 (0.7%)	0	1 (10%)	NS
50**–**59 years	10 (4.6%)	7 (4.8%)	0	1 (10%)	NS
60**–**69 years	31 (14.4%)	24 (16.4%)	3 (7.0%)	1 (10%)	NS
70**–**79 years	51 (23.7%)	38 (26.0%)	5 (11.6%)	3 (30%)	NS
80 plus years	121 (56.3%)	76 (52.1%)	35 (81.4%)	4 (40%)	<0.005^a^ <0.05^b^
No. of comorbidities (median, IQ range)	2 (1**–**3)	2 (1**–**3)	1 (1**–**2)	1 (1**–**3)	<0.001^a^
Any comorbidity	194 (90.2%)	135 (92.5%)	35 (81.4%)	8 (80%)	NS
Chronic heart disease	37 (17.2%)	42 (28.8%)	5 (11.6%)	2 (20%)	<0.05^a^
Chronic pulmonary disease except asthma	41 (19.1%)	33 (22.6%)	3 (7%)	1 (10%)	<0.05^a^
Asthma	9 (4.2%)	7 (4.8%)	2 (4.7%)	0	NS
Chronic kidney disease	20 (9.3%)	14 (9.6%)	2 (4.7%)	1 (10%)	NS
Diabetes mellitus	37 (17.2%)	25 (17.1%)	6 (14.0%)	2 (20%)	NS
Stroke	31 (14.4%)	24 (16.4%)	6 (14.0%)	1 (10%)	NS
Dementia	66 (30.1%)	36 (24.7%)	28 (65.1%)	0	<0.000005^a^ <0.001^b^
Cancer	34 (15.8%)	25 (17.1%)	2 (4.7%)	3 (30%)	NS
Myeloproliferative disorders	9 (4.2%)	7 (4.8%)	1 (2.3%)	0	NS
Liver disease	5 (2.3%)	5 (3.4%)	0	0	NS
Rheumatological disorders	11 (5.1%)	7 (4.8%)	0	1 (10%)	NS
Hypertension	30 (14.0%)	26 (17.8%)	2 (4.6%)	1 (10%)	NS

*χ*^2^ test with Yates correction for categorical variables and Mann−Whitney *U* test for continuous variables (age, number of comorbidities).

a Difference between hospitals and care homes.

b Difference between care homes and own home.

Of the 31(14%) patients less than 65 years, 20 were recorded as working and occupations included 2 NHS, 2 care sector and 1 transport sector staff.

Of 215 cases of COVID-19, 63 (29.3%) of infections were hospital acquired and a further 55 (25.6%) acquired in care homes.

## Discussion

This is the first comprehensive study of deaths, including individuals dying both in hospital and in the community, in the United Kingdom. Other studies have either looked at patients dying in hospital [4, 5, 7] or patients testing positive for SARS-CoV-2 in the community [6] or have been ecological studies [8].

The study uses the information required by a crematorium, under the law of England and Wales [9], before a person can be cremated, information which includes a brief clinical account to support the stated causes of death. Although unstructured text, official guidance on completion of the forms does exist [14] and this information can give a rounded picture of the circumstances leading to a death, similar to a clinical referral letter. Cremation forms, in this way, represent a rich source of information on the end of life and elements such as the type and appropriateness of care. This time-honoured and legally laid down process, can thus be used to promote health and prevent disease. To utilise better, this would require a degree of central organisation at regional or national level. The introduction of the Medical Examiner system, in England and Wales [15] may present an opportunity to do this but hitherto the focus has been almost entirely on patient satisfaction (or, more strictly, that of their relatives) and healthcare quality. Whilst important, this ignores the usages that mortality statistics have been put to, historically, to tackle other areas of public health such as health protection and health promotion, such as Clean Air Acts, following the 1953 London smog or the current tracking of COVID-19. In fact, better collation of mortality statistics and more extensive and systematic recording of clinical, pathological and risk factor data and linking those mortality statistics with other public data sources (e.g. cancer registries, prescribing data, hospital episode statistics, air quality data) would allow the contemporary quantification of several ‘big ticket’ current public health issues, other than COVID-19, such as, alcohol use, obesity, anti-microbial resistance and air pollution.

The denominator population, from which patients that use Cardiff Thornhill crematorium are drawn, is difficult to characterise exactly, as more than one crematorium serves the Cardiff Council area and equally cremations are accepted from other local authority areas. Cardiff itself has a population of 335 145 persons, according to the 2011 census and Thornhill crematorium users are thought broadly to reflect this.

COVID-19 deaths occurred, as in other studies, worldwide [16], mainly in the elderly (median age 82 years) with over half in those over 80 years old and with over 90% having pre-existing medical conditions. The proportion of men and women in the whole group did not differ significantly, unlike the other UK studies, restricted to deaths in hospital [4, 5, 7]. However, an excess of male deaths, in the subset of deaths occurring in hospital, was similar to these other UK studies. The odds of men dying in hospital was thus nearly twice that of women, even though they were of similar age with a similar number of comorbidities. This phenomenon is unexplained and it will be of interest to know if it is replicated in other settings where deaths in the community are also recorded.

Over half the infections, plausibly, were acquired in institutional care settings, of which nearly 30% were acquired in hospital. Concerns have been raised about hospital transmission in other UK settings but in the absence of official figures, the proportion has been estimated as 5–20% [17]. This inevitably poses the question of what proportion of these could have been prevented by more effective infection control procedures. It is particularly sobering to compare these percentages with the purported benefits of Non-Pharmaceutical Interventions (NPIs), as modelled by Imperial College, London [18] which were so influential in guiding government policy, in the UK, the United States and France, at the start of the pandemic. The biggest predicted percentage reduction in deaths, for any of the combinations of NPIs was also 50%. The implication of this is that more focussed interventions to prevent the introduction of SARS-CoV-2 and to control its spread, in healthcare settings, may have had the same potential to reduce deaths as the general social distancing and other costly measures that were, in the event, introduced. It poses the question whether the UK's high mortality, when compared with other European countries [19], has as much to do with its historically high hospital occupancy rates [20] and underfunding of the social care sector [21] as it does with the, widely blamed, delay in introducing lockdown [22]. Indeed, given the apparent ineffectiveness of the NPIs in keeping mortality down, in the UK, a focus on control of SARS-CoV-2 infection in hospitals, nursing and residential homes might have been a more effective approach in limiting deaths and certainly would not have resulted in the same social and economic disruption as lockdown and general social distancing. Going forward, this may still represent one of the most effective uses of testing and tracing teams, rather than attempting to extinguish spread in a wider community of people who would, largely, be expected to recover from the infection without mishap.

Cremation certificates represent a useful source of information on health problems locally and could, with greater co-ordination, contribute to a national picture. In a representative series of deaths, in persons, authorised for cremation, in South Wales, comprising both hospital and community deaths, persons over 80 with an average 2 comorbidities predominated. Although, unlike most other studies, there were similar proportions of men and women overall, men were more likely to die in hospital. Over half the infections were acquired in either hospitals or care homes with implications for the management of the pandemic, historically and in the future.

## Data Availability

The data set would, ordinarily, be shared on application, by email, to the authors.
